# Self-DNA in *Caenorhabditis elegans* Affects the Production of Specific Metabolites: Evidence from LC-MS and Chemometric Studies

**DOI:** 10.3390/molecules29204947

**Published:** 2024-10-19

**Authors:** Bruna de Falco, Adele Adamo, Attilio Anzano, Laura Grauso, Fabrizio Carteni, Virginia Lanzotti, Stefano Mazzoleni

**Affiliations:** 1 Dipartimento di Agraria, Università di Napoli Federico II, Via Università 100, Portici, 80055 Naples, Italy; bruna.defalco@unina.it (B.d.F.); attilio.anzano@unina.it (A.A.); laura.grauso@unina.it (L.G.); fabrizio.carteni@unina.it (F.C.); 2 Institute of Biosciences and BioResources, National Research Council, Via Pietro Castellino 111, 80131 Napoli, Italy; adele.adamo@ibbr.cnr.it

**Keywords:** self-DNA inhibition, model nematode, metabolite profile, bioactive metabolites, LC-MS analysis, PCA, OPLS-DA

## Abstract

The worm *Caenorhabditis elegans*, with its short lifecycle and well-known genetic and metabolic pathways, stands as an exemplary model organism for biological research. Its simplicity and genetic tractability make it an ideal system for investigating the effects of different conditions on its metabolism. The chemical analysis of this nematode was performed to identify specific metabolites produced by the worms when fed with either self- or nonself-DNA. A standard diet with OP50 feeding was used as a control. Different development stages were sampled, and their chemical composition was assessed by liquid chromatography–mass spectrometry combined with chemometrics, including both principal component analysis and orthogonal partial least squares discriminant analysis tools. The obtained data demonstrated that self-DNA-treated larvae, when arrested in their cycle, showed significant decreases in dynorphin, an appetite regulator of the nematode, and in N-formyl glycine, a known longevity promoter in *C. elegans*. Moreover, a substantial decrease was also recorded in the self-DNA-fed adults for the FMRF amide neuropeptide, an embryogenesis regulator, and for a dopamine derivative modulating nematode locomotion. In conclusion, this study allowed for the identification of key metabolites affected by the self-DNA diet, providing interesting hints on the main molecular pathways involved in its biological inhibitory effects.

## 1. Introduction

Metabolomics, the comprehensive analysis of endogenous metabolites in a biological system, allows researchers to gain insights into the physiological state, disease pathways, and environmental responses of an organism [1]. The global profiling of small molecules, including amino acids, lipids, and organic acids, gives information on the biosynthetic processes, complementing genomics and proteomics in the search for a systems-level understanding of biological systems [2]. Thus, metabolomics can be seen as a burgeoning tool in systems biology, providing an unparalleled opportunity to unravel the intricate metabolic profiles of organisms.

Recently, an increasing awareness of the generalized occurrence of environmental, free circulating, extracellular DNA (eDNA) is receiving higher attention in both ecology [3] and medicine [4]. The presence in the environment, as either substrate or diet, of the self-DNA of a species has been demonstrated to induce major inhibitory effects on individuals of the same species that are not, instead, affected by the exposure to heterospecific nonself-DNA [5,6,7,8,9].

The metabolomic approach has been recently applied to study the inhibitory effects on the growth of individuals by their own extracellular DNA (self-DNA). Significant effects have been reported in different model organisms: the plant *Arabidopsis thaliana* [10] and the insect *Drosophila melanogaster* [11].

A metabolomics study on *A. thaliana* showed a significant and generalized reduction in metabolic activities after exposure to the plant’s self-DNA, with molecular responses typical of stress conditions and the accumulation, in the exposed cells, of RNA components and precursors [10]. Further metabolomics research on *D. melanogaster* confirmed that self-DNA feeding reduced the amounts of all metabolites, particularly amino acids and N-acyl amino acids, known lipid signal mediators [11]. Moreover, in this study, an increasing amount of phloroglucinol was found after self-DNA exposure, and this was correlated to egg laying suppression. Also, in the same self-DNA-treated larvae, a significant increase in pidolate, a known intermediate in the γ-glutamyl cycle that is correlated to the blocking of insect oogenesis, was recorded [11].

In recent years, the nematode *C. elegans* has emerged as a compelling model for metabolomic studies, offering unique advantages for dissecting the dynamic interplay of small molecules within a living system [12]. In fact, *C. elegans*, with its transparent body, well-defined genetics, and short lifecycle, stands as an exemplary model organism for biological research [13]. The simplicity and genetic tractability of *C. elegans*, combined with its conservation of key metabolic pathways, make it an ideal system for investigating fundamental questions in metabolism, providing crucial insight [14].

Previous metabolomics studies on the nematode were used to explore the metabolic changes associated with different parameters such as aging, responses to environmental stressors, and the identification of biomarkers indicative of specific physiological states [15]. The integration of metabolomic data with other omics platforms enriches our understanding of the molecular basis of biological processes.

A very recent paper on *C. elegans* fed with self-DNA highlighted the setting of larval developmental defects and increased embryonic mortality, with magnification of the effects progressively increasing in subsequent generations [16]. In this model nematode, the observations of self-DNA impacts on egg fertility and fecundity clearly indicated an involvement of the germinal cell lines, whose mechanism is yet to be clarified. Moreover, the self-DNA inclusion in the nematode diet caused the onset of larval developmental defects and mortality, with a dramatic and progressive increase in such effects’ magnitude in the subsequent generations [16].

Here, we performed an untargeted chemical analysis of *C. elegans* extracts using an integrated approach of liquid chromatography–mass spectrometry (LC-MS), followed by chemometrics to obtain the metabolite profile and identify the components affected by self- and nonself-DNA-fed treatments.

## 2. Results and Discussion

### 2.1. C. elegans Treatments with Self- and Nonself-DNA and Extract Preparation

Previous work demonstrated the inhibitory effect in the progeny of worms exposed to self-DNA via their bacterial diet. The worms fed with a self-DNA bacterial genomic library showed developmental defects and germline DNA damage. Furthermore, we observed that the maturation to adulthood of eggs laid from F1 and F2 worms fed with self-DNA was delayed, compared to those fed on either control or nonself-DNA treatment [16].

**Figure 1:**
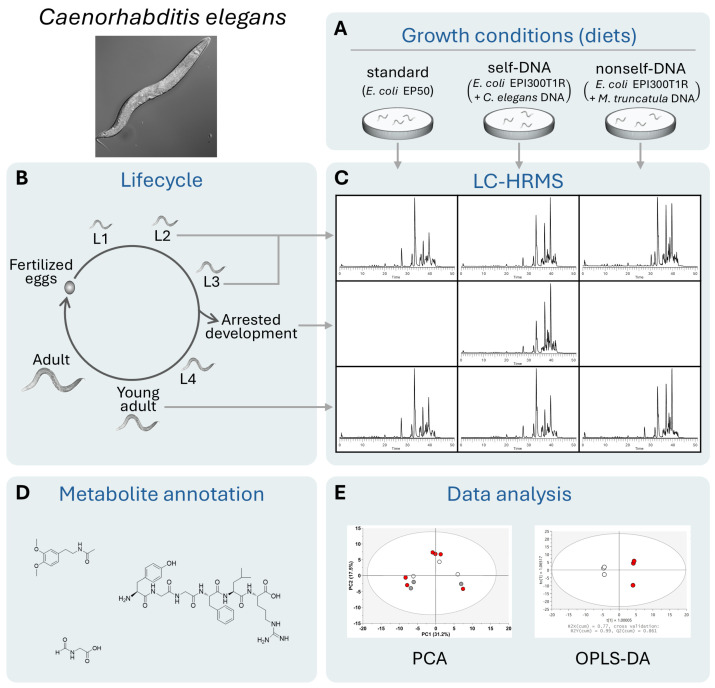
Schematic representation of the experimental work. Diets in different treatments are shown in (**A**). Stages of the life cycle are schematically represented in (**B**). All plots shown in (**C**–**E**) are examples of outputs of the corresponding analyses; the actual results will be presented in the following sections.

In order to investigate the metabolite profiles and the compounds affected by the exposure to either self- or nonself-DNA, we followed and collected samples from the IV generation (F4) of nematodes. Details on the growth conditions are reported in Germoglio et al. 2022 [16]. Figure 1 shows a schematic representation of the experimental workflow. In particular, the nematodes were fed with three different diets (Figure 1A) and sampled at three time points during their development: (1) combined L2 and L3 larvae, (2) larvae who arrested their development, and (3) young adults (Figure 1B). Liquid chromatography high-resolution mass spectrometry (LC-HRMS) and high-resolution tandem mass spectrometry (HR-MS/MS) were performed on all samples (examples of outputs are shown in Figure 1C), and the raw datasets were analyzed, after metabolite annotation (Figure 1D), using unsupervised principal component analysis (PCA) and orthogonal partial least squares discriminant analysis (OPLS-DA) (Figure 1E).

### 2.2. Compound Annotation by LC-MS Analysis

Data obtained by LC-MS were analyzed to obtain metabolite annotation through the untargeted metabolomics approach. The detection of metabolites was performed using Compound Discoverer 3.3 software (Thermo Fisher Scientific, Hemel Hempstead, UK). Metabolite annotation was performed by matching accurate masses and fragmentation patterns of detected peaks with those of analytical standards and online libraries, including the mzCloud fragmentation database. The confidence in metabolite identification was assigned from levels 1 to 4 following the Metabolomics Standards Initiative (MSI) guidelines. In this classification system, metabolites identified using *m/z*, RT, and/or MS/MS with reference standards were assigned to level 1. Metabolites putatively annotated using *m/z* and MS/MS from spectral libraries without reference standards were assigned to level 2, putatively characterized metabolite classes were level 3, and unknown metabolites were classified as level 4.

A total of 47 metabolites were annotated; 9 metabolites were identified using exact masses and the RT of authentic standards (level 1); 33 metabolites were putatively matched based on MS/MS fragmentation patterns (level 2); 4 metabolites were included in a class of compounds (level 3); and 1 compound remained unassigned (level 4) (Table 1).

### 2.3. Chemometric Analysis and Compound Quantification in Larvae

**Figure 2:**
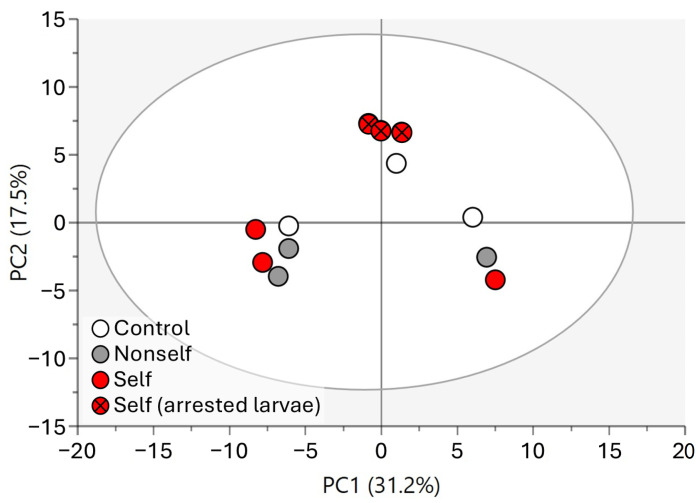
PCA score plot of larvae samples.

To evaluate differences in the metabolite profiles of larvae in the different treatments, the obtained data were analyzed by multivariate ordination. The resulting PCA score plot of larvae samples shown in Figure 2 accounted for 48.7% of the total variance, with component 1 explaining 31.2% and component 2 explaining 17.5%. Although there is no clear separation between the different treatment groups along the first axis, the data indicate a stronger clustering of the arrested larvae group that was clearly separated from the other treatments in the second component. However, no clear segregation became evident along the first principal component among the control, nonself-DNA treatment, and self-DNA larvae when these were not yet showing any phenotypic effect or reduction in growth rate.

**Figure 3:**
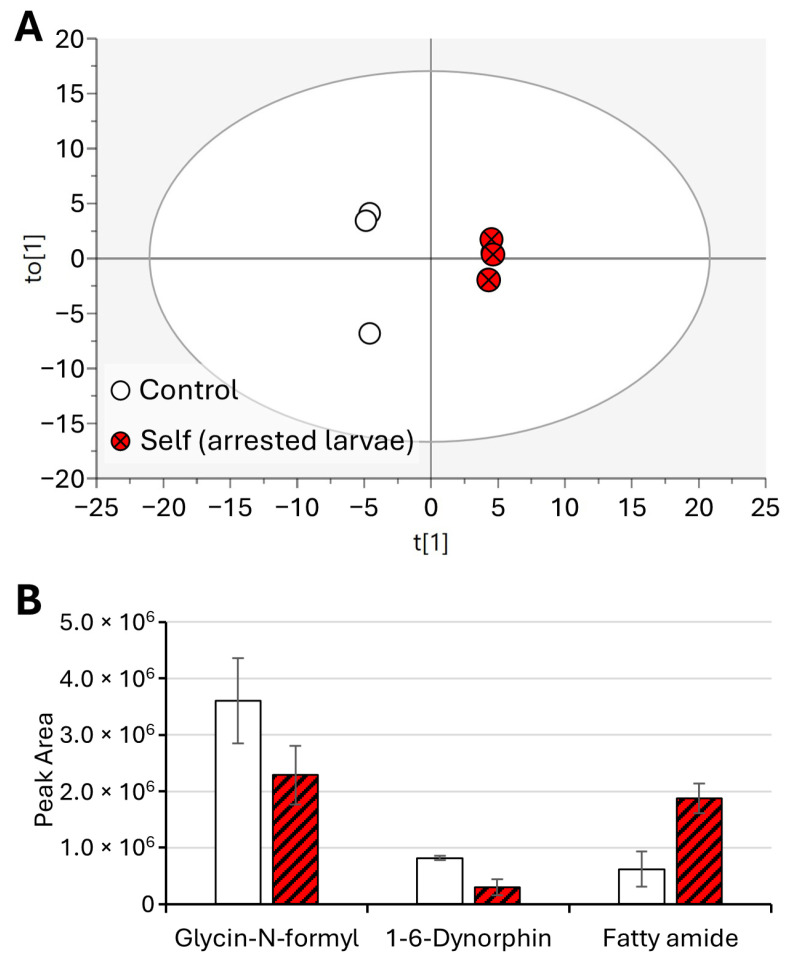
OPLS-DA of control larvae vs. self-arrested larvae (**A**) and bar plot of significantly different metabolites (**B**). Displayed data refer to the mean and standard deviation of three replicates.

To further investigate the significant perturbation between the control group of larvae samples and the larvae arrested after the self-treatment, OPLS-DA was employed (Figure 3A).

Cross-validation parameters indicate good predictive ability and model fitness, with values exceeding the recommended threshold of 0.50 for R2X (0.633), R2Y (0.99), and Q2 (0.94), suggesting that the model is robust [17]. Discriminative ions were determined using both multivariate (OPLS-DA) and univariate analysis (*p*-values). Variables with Variable Importance in Projection (VIP) > 1 in OPLS-DA models and *p* < 0.05 in the *t*-test were selected as variables responsible for the separation of samples in the models [18].

**Figure 4:**
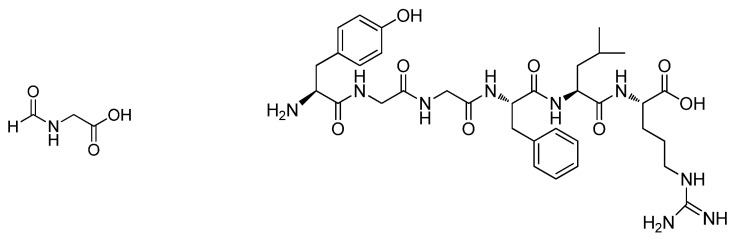
Chemical structures of Glycin-N-formyl (**left**) and 1-6-Dynorphin (**right**).

The bar plot obtained (Figure 3B) by peak integration showed significant decreases in glycin-N-formyl and 1,6-dynorphin (Figure 4). An increase in a fatty amide was observed. Thus, these compounds were selected as key variables responsible for the separation of samples (Figure 3B).

Deregulation of metabolism is known to be a hallmark of aging. As such, changes in the expression of metabolic genes and profiles of amino acid levels are features associated with aging animals. While the content of most amino acids decreases with age in *C. elegans*, glycine accumulates substantially. This finding was shown to be coupled with a decrease in gene expression of key enzymes for glycine catabolism. Additionally, glycine supplementation significantly extends the lifespan of *C. elegans*, and early adulthood is important for its health effects. Furthermore, glycine supplementation enhances specific transcriptional changes that are associated with aging. Glycine enters the methionine cycle, and its function in this pathway is correlated with its role in aging deceleration [19].

Dynorphin analogs are a class of opioid peptides arising from the cleavage of the protein prodynorphin by the enzyme proprotein convertase 2 (PC2). The cleavage is caused by the depolarization of a neuron containing prodynorphin stimulating PC2 processing and occurring within synaptic vesicles in the presynaptic terminal [20]. Several analogs have been isolated, named dynorphin A, dynorphin B, and α/β-neoendorphin [21]. Kappa opioid receptors (KORs) play a critical role in modulating dopamine, serotonin, and glutamate release in the central nervous system. Dynorphin is a peptide neurotransmitter processed from its precursor prodynorphin and is the endogenous ligand of the KOR [22]. Dysregulation of the dynorphin/KOR system has been implicated in several psychiatric diseases, including schizophrenia, depression, bipolar disorder, and drug addiction [22]. It is known that neuropeptides are also essential for the regulation of appetite [23]. Recently, it has been shown that *C. elegans* has an endogenous opioid system, which regulate feeding during starvation [24]. This is consistent with the finding that in many animals starvation increases exploration [25,26].

The fatty amide has been putatively identified as eicosaneneoyl amide, and is thus based on the C20:1 acyl group. It belongs to the class of Primary Fatty Acid Amides (PFAMs). Such metabolites are known to act as key signaling molecules in the mammalian nervous system, being able to bind many drug receptors. Their binding has been demonstrated to control many processes, including sleep, locomotion, and angiogenesis [27].

### 2.4. Chemometric Analysis and Compound Quantification in Adults

**Figure 5:**
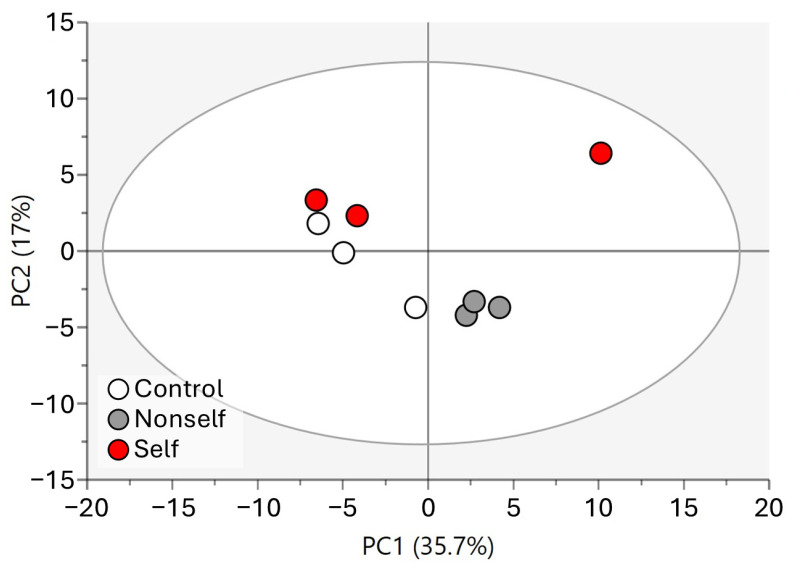
PCA of young adults (control vs. self vs. nonself).

Raw data from the LC-MS of adults in the different treatments were also analyzed by multivariate ordination. Figure 5 shows the PCA analysis of all young adults (control vs. self vs. nonself) of the IV generation. The first two principal components accounted for 52.7% of the original variation, with component 1 explaining 35.7% and component 2 explaining 17%. The ordination shows a segregation of both self- and nonself-DNA treatments compared to the control along the first axis, whereas the second component mostly separated the self-DNA treatment.

Furthermore, three different OPLS models were constructed, achieving significant group separation along both the predictive and orthogonal components.

**Figure 6:**
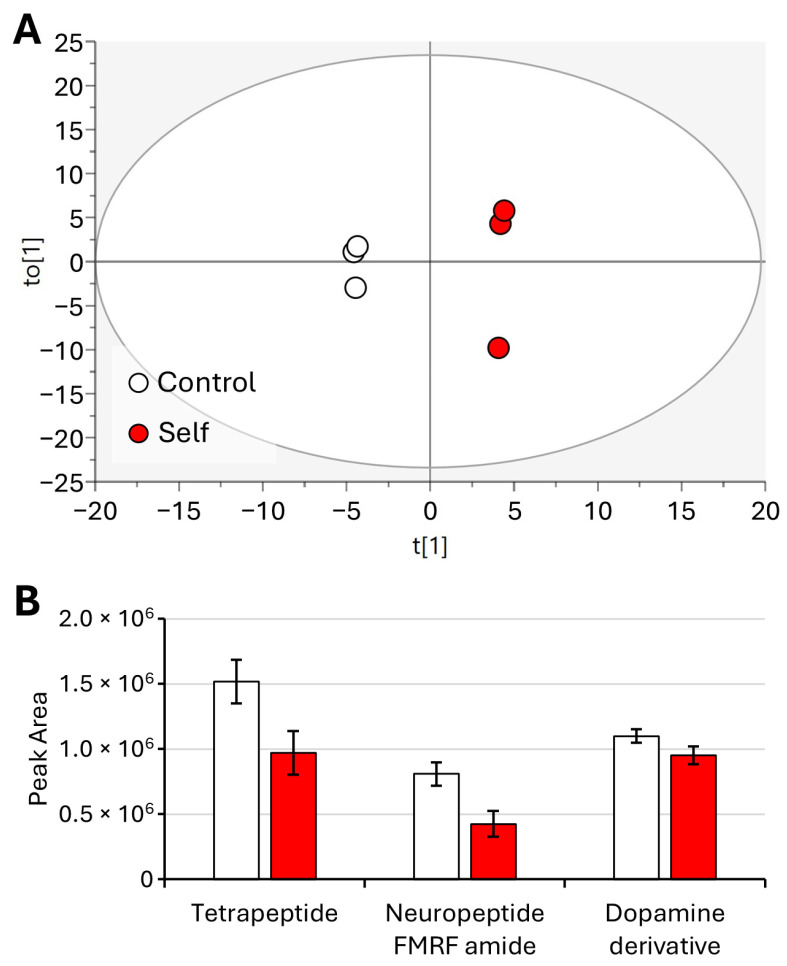
OPLS-DA of young adults (control vs. self) (**A**) and bar plot of significantly different metabolites (**B**). Displayed data refer to the mean and standard deviation of three replicates.

**Figure 7:**
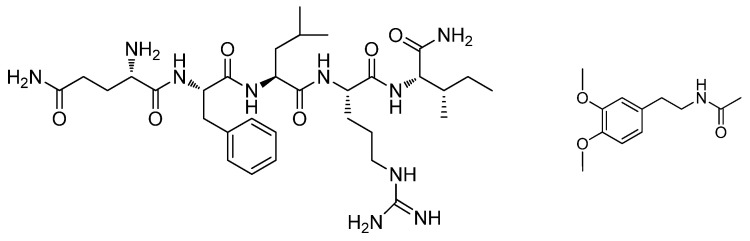
Chemical structures of neuropeptide FMRF amide (**left**) and dopamine derivative (**right**).

Figure 6 shows the data obtained by using OPLS-DA and quantitative analysis for young adults for control vs. self. Cross-validation parameters were excellent, with the cumulative R2X, R2Y, and Q2 values being 0.77, 0.99, and 0.86, respectively (Figure 6A). Three compounds were found to be significantly affected by the self-treatment, with decreasing amounts in the self-treated young adults (Figure 6B). The compounds were tetrapeptide, neuropeptide FMRF amide, and dopamine derivative (Figure 7).

FMRFamide-like peptides (FLPs) are evolutionarily conserved neuropeptides found in the animal kingdom that play critical roles in behavior, energy balance, and reproduction. Their sequences contain a common tetrapeptide core with additional amino acid units [28]. In nematodes, a diverse repertoire of extended peptides sharing the C-terminal RFamide motif was found. Some peptides show high sequence similarity to FMRFamide, suggesting homology to the tetrapeptide, and are therefore referred to as FMRFamide-related peptides (FaRPs) [29]. Liu et al. (2007) [30] studied the relationship between the nervous system, genes, and innate sexual behaviors. The authors demonstrated the function of the FMRFamide-like neuropeptide (flp) gene family in regulating male copulation and embryogenesis.

Recently, the identification of a dopamine receptor from *C. elegans* has been reported [31]. The authors showed the neurotransmitter dopamine as regulating locomotion and egg laying of the nematode. In detail, a cDNA encoding the *C. elegans* G protein-coupled receptor (CeDOP1) was cloned. The deduced amino acid sequence of the cloned cDNA shows high sequence similarities with D1-like dopamine receptors from other species. Dopamine showed the highest affinity (K(i) = 0.186 microM) for this receptor among several vertebrate and invertebrate amine neurotransmitters tested, suggesting that the natural ligand for this receptor is dopamine. Thus, dopamine is required for food sensation. In addition, dopaminergic neurons transduce the mechanosensory stimuli to modulate the locomotory rate [32].

**Figure 8:**
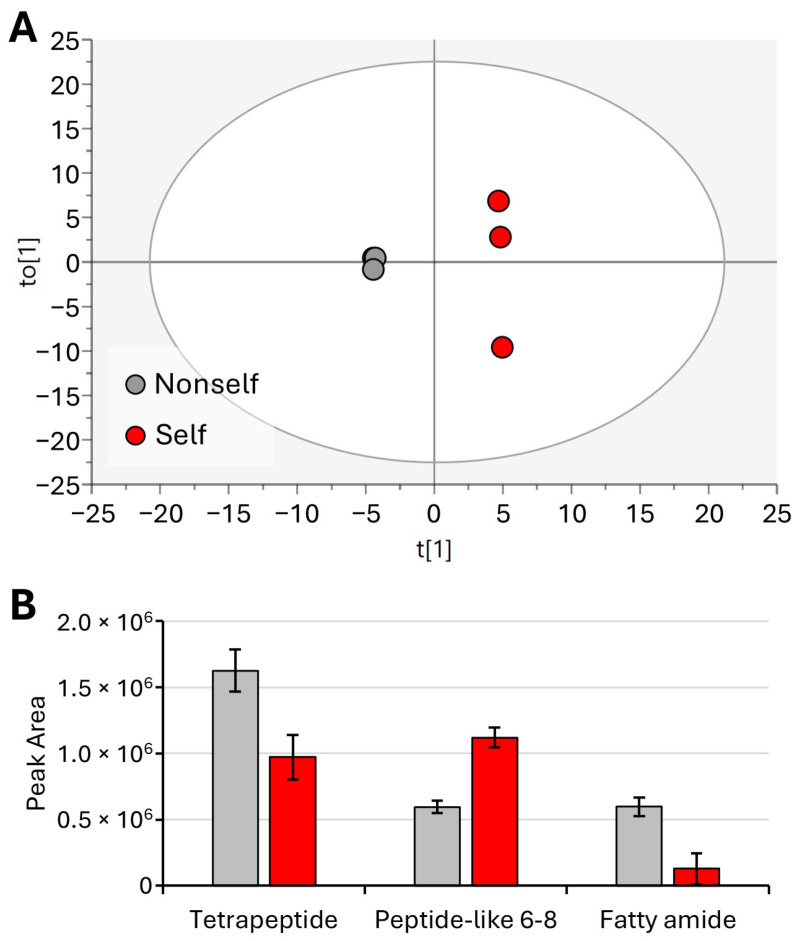
OPLS-DA of young adults (nonself vs. self) (**A**) and bar plot of significantly different metabolites (**B**). Displayed data refer to the mean and standard deviation of three replicates.

Proceeding with the comparison of metabolites that were significantly different in the different treatments, the nonself vs. self metabolite profiles were evaluated. Figure 8A shows the OPLS-DA model of nonself vs. self, where the cumulative R2X, R2Y, and Q2 values were 0.76, 0.99, and 0.90, respectively. Comparison of the treatments pointed to decreases in tetrapeptide and fatty amide and to an increase in 6–8 peptide-like compound (Figure 8B). It can be noted that the fatty amide compound that was found to increase in the arrested larvae shows an opposite trend in the case of adults that requires further investigation.

Neuropeptides play the crucial role in *C. elegans* of modulating a wide range of behaviors by allowing for changes in neuronal and synaptic signaling [33]. The finding of short-chain peptides, as compounds significantly differing between the nonself- and self-DNA treatments, indicates the involvement of neuronal and synaptic signals for recognition.

**Figure 9:**
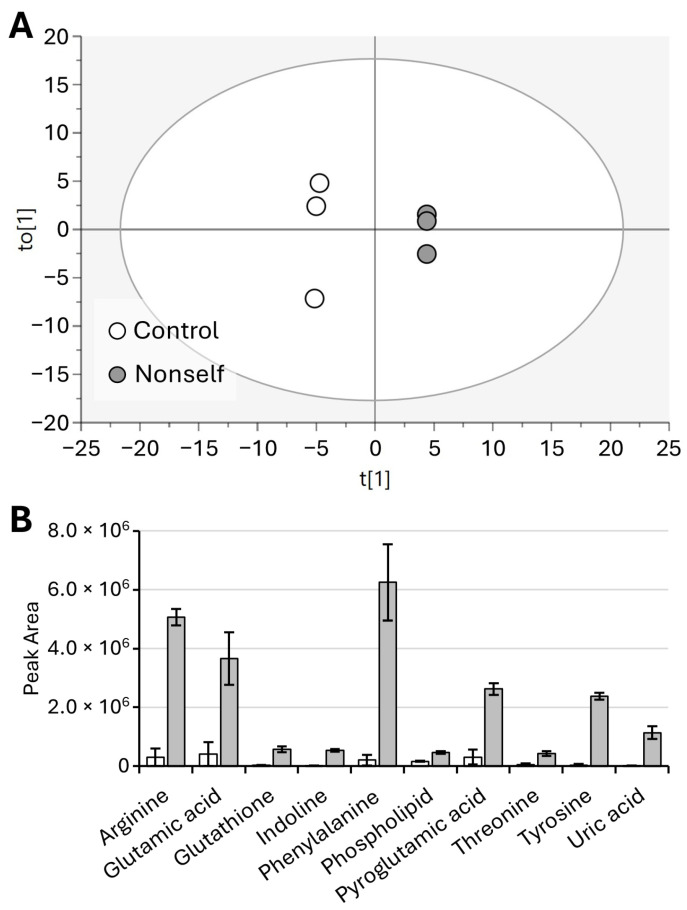
OPLS-DA of young adults (control vs. nonself) (**A**) and bar plot of significantly different metabolites (**B**). Displayed data refer to the mean and standard deviation of three replicates.

Finally, the OPLS-DA model of control vs. nonself-DNA treatment is shown in Figure 9A, where the cumulative R2X, R2Y, and Q2 values are 0.71, 0.99, and 0.88, respectively. Quantitative data on significantly altered metabolites responsible for the separation of samples in the OPLS-DA model are shown in the histogram (Figure 9B).

Major changes were observed in amino acids and heterocyclic compounds. In particular, the amino acids phenylalanine, tyrosine, arginine, glutamic acid, and threonine dramatically increased in the nonself treatment along with pyroglutamic acid and tripeptide glutathione. Moreover, the aromatic heterocyclic compound indoline, the heterocyclic uric acid, and a phospholipid were also found to increase.

## 3. Materials and Methods

### 3.1. Growth Conditions and Metabolite Extraction

The *C. elegans* strain wild type Bristol (N2) was provided by the Caenorhabditis Genetics Center. The *Escherichia coli* OP50 strain was used as the standard *C. elegans* food source. *E. coli* EPI300T1R is the bacterial strain hosting the fosmid libraries used in this study. The *C. elegans* and *Medicago truncatula* fosmid libraries were both produced in *E. coli* EPI300-T1R in the fosmid Copy Control vector pCC1FOS (fosmid copy number inducible by L-arabinose, chloramphenicol resistance). The *C. elegans* library (Source BioScience) consisted of 15,744 indexed bacterial clones (average insert size: 43.3 kb) distributed on 41 multiwell plates. The *M. truncatula* library (INRA-CNRGV) consisted of 68,352 clones (average insert size: 40 kb). It is a pooled library distributed on two multiwell plates in 178 pools. All the clones of the microbial libraries from each multiwell plate were grown at 37 °C overnight (total of 16 h) on Petri dishes containing chloramphenicol in solid agar LB. Subsequently, they were mixed and resuspended in glycerol (30%), in order to obtain different aliquots that were stored at −80 °C [16].

*C. elegans* was maintained on the nematode growth medium (NGM) agar plates seeded with the *E. coli* OP50 lawn at 20 °C for 3–4 days [34].

Each aliquot of frozen back pooled libraries was separately grown at 37 °C overnight (16 h) in LB supplemented with chloramphenicol (12.5 µg/mL) and L-arabinose (0.01%), and they were all mixed in order to obtain a combined pool representative of the whole genome. Finally, 1 mL of mixed bacteria with an OD600 of 12.5 nm was used as the food source on the NGM plate.

About 800 eggs synchronized with an alkaline hypochlorite solution from hermaphroditic adults fed OP50 (standard diet) were placed on NGM plates with L-arabinose (0.01%), inoculated with bacterial libraries (self-DNA, nonself-DNA) and with OP50 bacteria (3 petri plates for each food source), and incubated at 20 °C for 4 days.

After four days, the alkaline hypochlorite solution-synchronized eggs from the first generation (F1) were placed on fresh NGM plates containing L-arabinose and seeded with self or non-self or OP50 libraries. Every 4 days, about 800 eggs synchronized with alkaline hypochlorite solution were placed on fresh NGM plates containing L-arabinose and seeded with self or non-self or OP50 libraries, to obtain fourth-generation eggs (F4).

After 20 h from hatching, larvae (L2-L3 stage) from F4 were collected in H_2_Odd from three different food sources and were taken to dryness and thus stored at −80 °C. Furthermore, 100 young adults and 100 larval arrests were identified and collected in H_2_Odd 48 h after hatching and were taken to dryness and thus stored at −80 °C. Three biological replicates were run on each food source. The different stages were identified by morphological criteria and observed by Nomarski microscopy. In particular, unlike the larvae, young adults have a fully developed vulva, with two gonads still devoid of embryos. After about 10 h, the adult worms can be recognized by both gonads filled with developing embryos. *C. elegans* larvae and adults were extracted with a 1:1 solution of methanol/water, sonicated for 10 min, and centrifuged at 7000 rpm for 10 min at room temperature. The extract thus obtained was dried under vacuum and stored at −80 °C until analysis.

### 3.2. LC-MS Analyses

Liquid chromatography high-resolution mass spectrometry (LC-HRMS) and high-resolution tandem mass spectrometry (HR-MS/MS) were performed using a Thermo LTQ Orbitrap XL mass spectrometer (Thermo Fisher Scientific Spa, Rodano, Italy) coupled to a Thermo U3000 HPLC system. Metabolite separation was achieved by injecting 5 μL of sample on a Kinetex C18 column (5 μm, 50 × 2.1 mm, Phenomenex, Torrance, CA, USA), with a flow rate of 0.2 mL/min. The gradient elution, 0.1% formic acid in H_2_O (solvent A) and CH_3_OH (solvent B), was optimized as follows: 5% B 1 min, 5%–100% B over 40 min, hold 10 min. HR-MS and HR-MS/MS spectra were acquired in positive ion mode in the range of *m/z* 100–2000 with the resolution set to 60,000 and to generate data-dependent scans for identification. The MS parameters were as follows: spray voltage 4.80 kV, capillary temperature 285 °C, sheath gas rate 32.0 units N_2_ (ca. 150 mL/min), auxiliary gas rate 15 units N_2_ (ca. 50 mL/min). To prevent the formation of cluster ions while causing no actual fragmentation, source fragmentation was enabled using a mild potential of 35 V [35]. The MS/MS spectra of the selected ions were collected with collision-induced dissociation (CID) fragmentation, wideband activation mode, using the following parameters: isolation width ±3.00 Da, collision energy 35 units, activation Q 0.250 units, and activation time 30 ms [36].

### 3.3. Data Analysis and Metabolite Annotation

The raw LC-MS datasets were analyzed through the untargeted metabolomics approach. Compound Discoverer 3.3 SP2 software (Thermo Fisher Scientific, Hemel Hempstead, UK) was used for univariate analysis and metabolite annotation. Metabolite annotation was performed by matching accurate masses and fragmentation patterns of detected peaks with those of analytical standards and online libraries, including the mzCloud fragmentation database. The confidence in metabolite identification was assigned into levels 1–4 following the Metabolomics Standards Initiative (MSI) guidelines [37,38]. The retention time (RT) range applied was 0–45 min, the mass range was *m/z* 70–1050, the mass tolerance for peak picking and metabolite annotation was set to less than 5 ppm for both precursor and fragment ions, and the maximum retention time shift was 0.25 min. Multivariate data analysis was conducted using SIMCA18 (Sartorius, Gottingen, Germany). The datasets were mean-centered and Pareto scaled prior to model inclusion. Logarithmic transformation was applied to minimize the impact of both noise and high variance among variables [39].

The data were first explored using unsupervised principal component analysis (PCA). Subsequently, orthogonal partial least squares discriminant analysis (OPLS-DA), a supervised projection-based analysis, was performed to maximize the variation between groups and to determine variables contributing to this variation. Scatter plots, loading plots, and Variable Importance in Projection (VIP) score plots were generated and interpreted to identify significantly altered metabolites between the control and treatment groups (self and nonself-DNA). Metabolites with VIP scores greater than 1 were considered strong contributors. The quality of models was validated through cross-validation using the leave-one-out method, assessing the goodness of fit of the model (R2X) for PCA, (R2Y) for OPLS-DA, and predictive ability (Q2) values [17]. A *p*-value less than 0.05 was considered to define statistical significance.

## 4. Conclusions

The untargeted metabolomics approach on polar and apolar extracts of *C. elegans* allowed the characterization of the metabolite profile, and the further identification and quantification of several primary and secondary metabolites by using a combination of LC-MS and chemometrics analyses. Several compounds were found at significantly different levels in worms treated with self- and nonself-DNA-enriched diets compared to the control.

Interestingly, the changes in the metabolite profile observed in this work on *C. elegans* were found to be consistent with similar studies on other different model organisms. On one hand, a generalized depression in protein synthesis with accumulation of RNA constituents has been reported in the model plant *Arabidopsis thaliana* [10] when treated with extracellular self-DNA. Also, in *C. elegans* a depression in the general metabolite profile was found in this study, with a reduced number of detectable spectral signals in the self-DNA-fed worms. In particular, a significant decrease in glycine in the self-DNA-treated arrested larvae was found. These data agree with the observation that glycine supplementation significantly extends the lifespan of *C. elegans*, enhancing animal health [40]. On the other hand, the significant stimulation of amino acid production after exposure to nonself-DNA has also been reported in *A. thaliana* [10].

Noteworthily, the finding of a decreased amount of fatty amide in the nematode adults treated with self-DNA is interesting because these compounds have also been reported in *D. melanogaster* for being potent cell signaling lipids [40], involved in the inactivation of neurotransmitters, cuticle sclerotization, and melatonin biosynthesis [41,42,43]. In the case of the *D. melanogaster* study [11], a significant decrease in such metabolites was observed in self-DNA samples as well, indicating the partial inactivation of these metabolic processes. On the other hand, in this study, we found that the arrested larvae showed higher levels of this metabolite. This might possibly be related to an early compensatory reaction to the self-DNA treatment, ending up at the later adult stage with the onset of the inhibitory effect. However, this specific point and the time dynamics between early and late responses of metabolic profiles will require further investigation.

In conclusion, this work, while confirming on another important model organism the general occurrence of the inhibitory effect by extracellular self-DNA already demonstrated in plants, insects, and yeast, highlighted a noticeable reduction in neuropeptides. Interestingly, in the arrested larvae a significant decrease in the opioid peptide 1,6-dynorphin was found. Apart from the involvement of dynorphin in several psychiatric and mental diseases [22], it was recently also reported that it has an essential role as an appetite regulator [23]. Moreover, concerning young adults, the decrease in FMRF amide-like peptides and the related tetrapeptide in worms after self-DNA treatments points to the involvement of neuronal transmission inhibiting worm reproduction and embryogenesis [30].

Finally, the decrease in a dopamine derivative has also been found in young adults treated with self-DNA. It is known that such compounds act in *C. elegans* by regulating locomotion and egg laying. Dopamine is required for food sensing, and dopaminergic neurons modulate the locomotory rate [31,32].

In conclusion, this work provides further insight into the effect of self-DNA on the model organism *C. elegans.* In plants, several papers have demonstrated either inhibitory [5,6,44] or stimulatory [45,46] effects of extracellular DNA fragments of either self or nonself sources, respectively. Such effects were reported after exposure to fragments sized in a range between 100 and 3000 bp. However, the inhibitory effect was shown to vary according to fragment size. For example, the activation of MAPKs after treatment with self-DNA occurred with fragments sized less than 700 bp, while a range of 700–1000 bp was found to not be active [44]. Similarly, inhibition of root growth was not observed with genomic DNA while sonication to a size lower than 2000 bp activated the effect [5]. In this work, we are focusing on a different biological system, an animal instead of plants. In this case, the model nematode was fed with bacteria with DNA inserts averaging in size over 40 kb. After feeding, the bacteria are digested, and so their biochemical compounds, including their DNA, are degraded before their assimilation in the gut, likely reducing their size to lower dimensions, comparable to the range reported to be active in plants. Future work could further investigate the digestion and assimilation processes in the nematode gut.

Finally, ongoing research is focusing on nematode motility and feeding behavior in association with the chemical characterization of metabolites involved in the self-DNA inhibitory effect. Further investigation is required to better clarify the molecular pathways involved in this phenomenon, which demonstrates once more the functional impact of extracellular self-DNA.

## Figures and Tables

**Table 1:** Metabolites identified in the analyzed extracts.

Annotation	Formula	RT [min]	*m/z*	Reference Ion	∆ Mass [ppm]	Id. Level
Glycin-N-formyl *	C_3_H_3_NO_3_	29.28	101.00830	M^+^	−0.38	2
L-(+)-Valine	C_5_H_11_NO_2_	0.79	118.08622	[M + H]^+^	−0.33	1
Threonine *	C_4_H_9_NO_3_	0.79	120.06540	[M + H]^+^	−0.32	2
Indoline *	C_8_H_9_N	2.32	120.08082	[M + H]^+^	0.34	2
D-(+)-Pyroglutamic acid	C_5_H_7_NO_3_	0.80	130.04981	[M + H]^+^	−0.04	1
L-(+)-Leucine	C_6_H_13_NO_2_	1.30	132.10187	[M + H]^+^	−0.26	2
Pipecolic acid	C_6_H_11_NO_2_	0.72	147.11288	[M + NH_4_]^+^	0.37	2
DL-Glutamic acid *	C_5_H_9_NO_4_	0.79	148.06043	[M + H]^+^	−0.02	1
3-Methylsulfolene	C_5_H_8_O_2_S	0.86	150.05826	[M + NH_4_]^+^	−0.55	2
L-Methionine	C_5_H_11_NO_2_S	0.94	150.05843	[M + H]^+^	0.67	2
DL-Histidine	C_6_H_9_N_3_O_2_	0.76	156.07680	[M + H]^+^	0.33	2
Fucosamine	C_6_H_13_NO_4_	0.80	164.09181	[M + H]^+^	0.49	2
DL-Phenylalanine *	C_9_H_11_NO_2_	2.35	166.08637	[M + H]^+^	0.72	1
Uric acid *	C_5_H_4_N_4_O_3_	1.18	169.03575	[M + H]^+^	0.8	2
Aminolevulinic acid	C_5_H_9_NO_3_	0.81	173.09230	[M + ACN + H]^+^	0.95	2
Unknown *	C_3_H_2_N_4_O_3_S	0.93	174.99155	[M + H]^+^	−2.82	4
N~2~-Acetyl-L-ornithine	C_7_H_14_N_2_O_3_	0.78	175.10774	[M + H]^+^	0.13	1
DL-Arginine *	C_6_H_14_N_4_O_2_	0.74	175.11912	[M + H]^+^	0.96	1
4-Methyleneglutamine	C_6_ H_10_ N_2_ O_3_	0.79	176.10300	[M + NH_4_]^+^	0.14	2
DL-Citrulline	C_6_H_13_N_3_ O_3_	0.82	176.10312	[M + H]^+^	1.09	1
DL-Tyrosine *	C_9_H_11_ NO_3_	1.37	182.08135	[M + H]^+^	0.99	1
N(1)-acetylspermidine	C_9_H_21_N_3_O	0.74	188.17575	[M + H]^+^	0.06	2
α-Aminoadipic acid	C_6_H_11_NO_4_	0.79	194.10240	[M + H + MeOH]^+^	0.57	2
N-Acetyl-L-histidine	C_8_H_11_ N_3_O_3_	0.79	198.08754	[M + H]^+^	1.13	2
N-(3.4-Dimethoxyphenethyl) acetamide *	C_12_H_17_NO_3_	0.72	224.12828	[M + H]^+^	0.73	2
N-Acetylcystathionine	C_9_H_16_N_2_O_5_S	1.59	265.08543	[M + H]^+^	0.62	2
Adenosine	C_10_H_13_N_5_O_4_	1.85	268.10422	[M + H]^+^	0.7	1
2-Amino-1.3-hexadecanediol	C_16_H_35_ NO_2_	25.65	274.27418	[M + H]^+^	0.45	2
(9Z)-9-Octadecenamide	C_18_H_35_NO	32.15	282.27942	[M + H]^+^	0.99	2
Oleic acid	C_18_H_34_O_2_	34.19	283.26340	[M + H]^+^	0.85	2
Argininosuccinic acid	C_10_H_18_ N_4_O_6_	0.83	291.13004	[M + H]^+^	0.43	2
Methyl (9E)-9-octadecenoate	C_19_H_36_O_2_	34.56	297.27921	[M + H]^+^	1.34	2
Glutathione *	C_10_H_17_N_3_O_6_S	0.86	308.09130	[M + H]^+^	0.72	2
Fatty amide *	C_20_H_39_NO	33.37	310.31053	[M + H]^+^	0.28	3
Eicosapentanoic acid	C_20_H_30_O_2_	31.70	325.21451	[M + Na]^+^	1.42	2
Erucamide	C_22_H_43_NO	34.49	338.34205	[M + H]^+^	0.9	2
Tetrapeptide *	C_19_H_38_N_6_O_4_	29.82	415.30330	[M + H]^+^	1.37	3
Tripeptide-like *	C_17_H_25_N_3_O_9_	20.66	416.16670	[M + H]^+^	0.83	3
1-Myristoyl-2-hydroxy-sn-glycero-3-PE	C_19_H_40_NO_7_P	29.46	426.26204	[M + H]^+^	1.24	2
(2R)-1-(Octanoyloxy)-3-(phosphonooxy)-2-propanyl decanoate	C_21_H_41_O_8_P	31.05	435.25079	[M + H − H_2_O]^+^	0.47	2
Glycochenodeoxycholic acid	C_26_H_43_NO_5_	27.63	450.32214	[M + H]^+^	1.64	2
(2R)-3-{[(2-Aminoethoxy)(hydroxy) phosphoryl]oxy}-2-hydroxypropyl (9Z)-9-hexadecenoate *	C_21_H_42_NO_7_P	29.65	452.27783	[M + H]^+^	1.48	2
LysoPC(18:3(9Z.12Z.15Z))	C_26_H_48_NO_7_P	29.88	518.32428	[M + H]^+^	0.32	2
1-[(8Z.11Z.14Z)-icosatrienoyl]-sn-glycero-3-phosphocholine	C_28_H_52_NO_7_P	31.46	546.35559	[M + H]^+^	0.32	2
Neuropeptide FMRF amide *	C_32_H_54_N_10_O_6_	28.86	675.42920	[M + H]^+^	−1.27	2
1-6-Dynorphin *	C_34_H_49_N_9_O_8_	31.84	711.37065	[M + H]^+^	0.41	2
6–8 peptide-like residues *	C_30_H_44_N_12_O_10_	30.07	733.33700	[M + H]^+^	−0.83	3

* Metabolites that were significantly altered between treatments.

## Data Availability

The data used to support the findings of this study are included within the article.
